# A Scientometric Analysis and Visualization of Research on Parkinson's Disease Associated With Pesticide Exposure

**DOI:** 10.3389/fpubh.2020.00091

**Published:** 2020-04-07

**Authors:** Chaoyang Liu, Zehua Liu, Zhentao Zhang, Yanan Li, Ruying Fang, Fei Li, Jingdong Zhang

**Affiliations:** ^1^Research Center for Environment and Health, Zhongnan University of Economics and Law, Wuhan, China; ^2^Department of Neurology, Renmin Hospital of Wuhan University, Wuhan, China; ^3^School of Information and Safety Engineering, Zhongnan University of Economics and Law, Wuhan, China

**Keywords:** Parkinson's disease, pesticide exposure, knowledge mapping, bibliometric, citespace

## Abstract

The etiology and pathogenesis of Parkinson's disease (PD) have not yet been clearly described. Both genetic and environmental factors contribute to the onset and progression of PD. Some pesticides have been demonstrated to be associated with PD by many previous studies and experiments, and an increasing number of researchers have paid attention to this area in recent years. This paper aims to explore the knowledge structure, analyze the current research hot spots, and discuss the research trend through screening and summarizing the present literature. Based on 1767 articles from the Web of Science Core Collection and PubMed database, this study carried out the analysis from the keywords, cited references, countries, authors, and some other aspects by using Citespace. The hot topics, valuable articles, and productive authors in this research field could be found after that. To the best of our knowledge, this is the first study to specifically visualize the relationship between pesticide exposure and PD, and forecast research tendency in the future.

## Introduction

Parkinson's disease (PD) is one of the most common neurodegenerative diseases, mainly affecting the middle-aged and the elderly. In America, the incidence of PD among people over 65 years old is about 1.6%, with 50,000 to 60,000 new cases diagnosed annually (1). With the global trends in aging, the number of people affected by PD is expected to increase significantly. China has the largest number of PD patients in the world, which are estimated to grow to 5 million in 2030 (2). PD patients in France are estimated to be 260,000 in 2030, corresponding to an increase of 65% compared with 2010 (3). Consequently, the cost of PD will skyrocket. It is assumed that the annual cost of PD in the US alone would exceed $50 billion by 2040 (4).

Pathological studies of several rare hereditary parkinsonism cases found no Lewy body or deposition of α-synuclein (5). However, the main neuropathological features of PD are the progressive loss of dopaminergic neurons in the substantia nigra pars compacta (SNpc) (6). Sporadic PD is also characterized by the presence of Lewy bodies and Lewy neurites, which consist primarily of misfolded and aggregated protein α-synuclein in the remnant neurons (7–9). Mitochondrial dysfunction, oxidative stress, protein aggregation, and inflammation play indispensable roles in the pathogenesis of PD (10), each of which could lead to the degeneration of dopaminergic neurons. As a chronic progressive neurodegenerative disorder, the clinical manifestations of PD include many motor symptoms, such as static tremor, bradykinesia, and rigidity (11). In addition, there are some non-motor symptoms in PD patients, including depression, cognitive impairments, and gastrointestinal dysfunction, which may appear years or even decades before motor symptoms (12).

The features of PD have been mentioned in Indian texts for thousands of years, and the full syndrome was described in the early nineteenth century (13). However, until now, the etiopathogenesis of PD has not been clearly understood. The current treatment methods have limited efficacy on the loss of dopaminergic neurons but only alleviate the symptoms partially and unsustainably (14). In recent years, the etiology of PD has been discussed in many studies, where aging, genetic, and environmental factors are the main concerns (15).

The environmental hypothesis and the genetic hypothesis are frequently discussed in the etiology of PD. So far, 23 loci and 19 disease-causing genes related to PD have been found. But it is controversial to classify genetic factors as the primary cause of PD, as the genetic factors can only explain a few cases, and 90% of PD patients have no family history (16). At present, it is widely accepted that genes, environmental toxins, and other factors, such as aging and lifestyle, combine to cause PD. From the descriptive observational epidemiology, Brown found a relatively consistent relationship between pesticide exposure and PD, and this relationship was the most obvious after long-term exposure to pesticides (17).

The association between environmental factors and PD was first proposed in 1982, when patients poisoned with 1-methyl-4-phenyl-1,2,3,6-tehydropyridine (MPTP) showed symptoms of acute PD and were relieved by the standard PD therapeutic medication at that time (18). Hereafter, the environmental hypothesis of PD has attracted more attention. Chemicals and drugs with similar toxicological profiles have been associated with PD in many studies, typically the intensive study of the pathogenesis of PD induced by MPTP. After crossing the blood–brain barrier (BBB) freely, MPTP is converted into the proximate toxin, MPP+ (1-methyl-4-phenylpyridinium), by glial monoamine oxidase B (MAO-B). Then, MPP+ is excreted from astrocytes via organic cation transporter-3 (OCT3) and absorbed by presynaptic dopamine transporter on dopaminergic nerve endings (19). Not being sequestered into vesicles by the vesicular monoamine transporter, MPP+ is absorbed by mitochondria and inhibits Complex I, interfering with the process of oxidative phosphorylation, thereby stressing the cell by reducing the synthesis of ATP (20). In this process, cells might produce free radicals resulting in lipid peroxidation, which damages cellular membrane and stress mitochondria, leading to bioenergy failure and cell death through positive feedback (21). Moreover, rotenone, a functional analog of MPTP, and paraquat, a structural analog of MPTP, have also been proven by many researchers to be significantly correlated with PD. Due to basically the same pathogenesis, both of them have inhibitory effects on mitochondria in dopaminergic neurons, giving rise to a series of toxic effects (14).

Rotenone, paraquat, organochlorine, organophosphate, and other pesticides, especially the first two chemicals, have been extensively concerned in the study of PD associated with exposure to pesticides. Among the 1767 literatures analyzed in this study, there were more than 400 articles related to paraquat and over 900 articles related to rotenone. Therefore, the studies on these two pesticides indicate more reliable scientific evidence, and rotenone-induced animal models of PD have also been used in the research and development of many PD drugs. The papers screened in this study were published from 1986 until 2019, and all of them could be classified into three groups: the first group is the review papers, which summarize various environmental factors such as pesticides, metals, lifestyle, genes, and other pathogenic factors. Based on the previous findings, Elbaz, et al. analyzed many potential pathogenic factors and described the pathogenic mechanisms of several typical pesticides in detail (22). The second category of literatures confirmed and analyzed the pesticide-induced PD, namely, exploring the pathogenesis through the animal models induced by pesticides and clinical case studies, or discussing the correlation of pesticide exposure and PD in epidemiological studies. Zhang et al. studied the typical features of PD lesions induced by different doses of rotenone subcutaneously injected into rats, so as to identify the optimum dose of subcutaneous rotenone for establishing a model of PD (23). The third group is about the exploration of the combined effects of genes and pesticides, which is usually aimed at abnormal proteins related to mutations in DNA under the pesticide exposure and studies the changes in its mechanism of action on cells under the genetic mutations in PD-associated proteins. In the study of Paul et al., the combined pathogenicity of paraquat and maneb was utilized to investigate the effects of three expression-altering NFE2L2 SNPs and four PPARGC1α SNPs on the risk and pathogenesis of PD (24).

Citespace can measure and analyze scientific publications statistically. In addition, this software can realize information visualization by generating various node-type networks. Created by Chen in early 2004 (25), Citespace usually works as an auxiliary tool for researchers to evaluate the knowledge base, hot spots, key points, as well as research trend. Recently, its function in literature analysis has played a vital role in all fields of technology and science (26). However, Citespace can only outline the general situation of the research field, but cannot present detailed information of the literature. Therefore, based on the analysis results of Citespace and critical reading of the literature, this study will further sort out the literature systematically in this field.

In this study, we collected and screened the literature related to the research on PD associated with exposure to pesticides, so as to form a unique literature library. Then, we used Citespace to conduct statistic computation of the information in the literature and further generated visual results with different node types such as keyword, country, author, and some others. Thus, this study aims to (i) identify collaborative networks and major scholarly communities in the research field of pesticides associated with PD; (ii) analyze the research status and hot spots, especially the specific type of pesticide inducing PD, and the high co-reference articles with creative ideas and reliable experiment findings; and (iii) discuss and summarize the research trend, including the study of the combined effect of potential pathogenic factors in the future.

## Materials and Methods

### Introduction to Scientometrics and Visualization in Citespace

Through the scientometric analysis, Citespace could count the kinds of records in the literature, such as authors, countries, keywords, and references. As users can set up parameters like time slicing, term source, and threshold, it will generate varied results (27, 28). Furthermore, Citespace could visualize the results of analysis, in which the color represents the year, the size of the node represents the amount or frequency, and the thickness of the link corresponds to the strength of association (29). Specifically, for example, the color of a link represents the year of the first relationship established between two nodes (e.g., the year in which two authors first cooperated in terms of the author co-citation network). Therefore, the quality of different items and their relationship with each other could be reflected clearly by nodes and links in the visualization results (30). In addition, Citespace can analyze the burst of items to find out the keywords with long-term burst and high strength, and it can cluster the items named by optional algorithms like log-likelihood ratio (LLR) (31). Compared with other visualization tools, Citespace improves the clarity and interpretability of visualizations, which helps users reduce cognitive burden to find vital trends and pivotal points in a knowledge structure (25, 32, 33).

### Data Collection

This study aims to review the articles about pesticide exposure-induced PD, and the types of pesticides need to be as many as possible, so we added many specific pesticides as keywords for searching. The time span should be as long as possible. In this study, Parkinson's disease and Pesticide were searched jointly as terms of theme, as follows: Rotenone, Paraquat, Organophosphate, Organochlorine, Dithiocarbamate, Carbamate, and some other typical pesticides with PD were added respectively for joint retrieval. A total of 4,530 articles were obtained from the Web of Science (WOS) Core Collection and PubMed before 26 February 2019. After screening by previously selected themes, 1,767 English articles that met the requirements of this study constituted a proprietary literature library, and all of them were published from 1986 to 2019. For the literature not included in this study, some of them were duplicate publication, and some of the articles simply mentioned pesticides or PD in the abstract, which did not meet the purpose of this study.

### Data Analysis

After importing the literature into the 5.3.R4 SE version of Citespace, the overall time span adopted was from the year 1986 to 2019, with a 3-year time slice, and the top 50 of the most cited or occurred items were selected from each slice. The Term Source included Title, Abstract, Author, Keywords, and Keyword Plus. Node Types were selected as Reference, Keyword, Country, Institution, Author, and Cited Journal, and the data would be visualized one by one.

Therefore, the mainly important procedures set up in scientometric analysis with Citespace are as follows: (1) import the articles and adjust the data format, (2) adjust the time slicing, (3) limit the term sources, (4) select the node types, and (5) set up the selection criteria on each slice. In the practical operation, the node types would change for different analysis aims, and the other procedures are usually kept constant. The analysis results of co-occurred keywords and co-referred references could show the content and research direction of the articles, while the others could help us identify the current situation and find out the most productive teams in this field.

In the visualization result of keyword co-occurrence, keywords with high frequency could be presented, and time slicing of their occurrence and the strength of their collaboration would be shown by nodes and links. In the process of this analysis, the burst detection reveals hot spots and the research trends. Moreover, the co-citation analysis identifies the highly cited reference and the situation of being referred in different periods, with the association between references shown in the networks. Furthermore, the cluster analysis and timeline views help to recognize the structure and evolution of this research (34).

## Results

### Analysis of Publications

**Figure 1:**
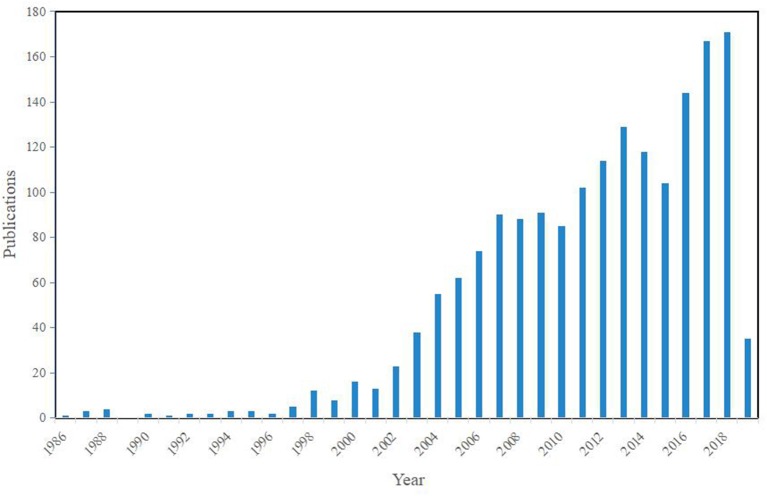
Temporal distribution of the publications.

From 1986 to 2019, the distribution of annual publications is shown in Figure 1. Before 2002, the number of publications about pesticide-induced PD was small, with less than 20 articles published every year, revealing that this research field was at the preliminary stage. However, there was a breakthrough after 2003, and the yearly outputs showed a rising trend although a slight stagnation appeared in the period from 2007 to 2010 and from 2014 to 2015. Overall, the outputs increased continuously nearly 30 years, with the appearance of some reviews and the new findings in some typical pesticides, as well as the innovation of methods and the type of pesticides. Nevertheless, the average number of publications is 53.55, and even 122.5 in the past decade, which has not reached a high level, suggesting that this field still lacks attention and important breakthroughs.

### Analysis of Co-occurrence Keywords

**Figure 2:**
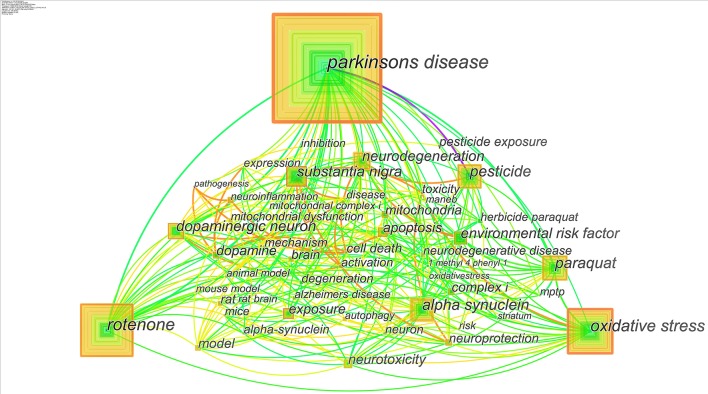
Joint mapping of co-occurrence keywords.

As shown in Figure 2, the analysis, utilizing “keyword” as node type, is visualized after searching the titles, abstract, and keywords of the articles through Citespace. Every square node represents a keyword, and the line between two nodes means the co-occurrence. In this study, there are 101 keywords, and the number of their co-occurrence reached 437. The lines and the square rings forming the nodes own different colors and thicknesses. Twelve kinds of colors respectively correspond to different time slices, containing 3 years per slice and spanning from 1986 to 2019 in this study. In Figure 2, the purple area denotes that the keywords exist from 1986 to 1988, the blue area represents the earlier years, the green and the yellow areas correspond to the middle and later years, and the red zone means that the keywords occur in 2019. The thickness of the square ring is proportional to the frequencies, and the thickness of lines depends on the intensity of their collaboration links.

Figure 2 shows the popular research topics including PD, Rotenone, Paraquat, Oxidative Stress, α-Synuclein, Dopaminergic Neuron, Substantia Nigra, and Neurodegeneration. Frequently appearing in every time slice, these keywords are also highly relevant to other research topics due to their links with each other. Among them, the appearance of Rat, Mice, and Model could reflect the importance of animal models in the study of pesticide-induced PD. The study in molecular mechanism is hot as the frequent occurrence of Oxidative Stress and α-Synuclein. Moreover, the most extensively studied environmental toxins such as Rotenone, Paraquat, Maneb, and MPTP could also be found in the visual result.

To show the frequency and centrality of the main keywords clearly, the top 20 keywords with the most citations are summarized in Table 1. The centrality of a node could quantify the importance of its position in a network (25). Searching through Citespace, the keywords that appeared in the articles in the 1990s were not in a high frequency as expected for there were little co-occurrence. Most of the highly cited keywords have occurred since 2004, which is consistent with the rising publications (Figure 1). Consequently, we could find that Rotenone and Paraquat are the hottest topics and often used as drugs to induce the animal model of PD. Besides, Paraquat is usually combined with Maneb for inducing PD in mouse model.

**Table 1:** Top 20 keywords with high citation.

**Frequency**	**Keyword**	**Centrality**	**Year**
798	Parkinson's disease	0.50	1986
437	Rotenone	0.12	1986
337	Oxidative stress	0.23	2004
221	Pesticide	0.04	1986
220	Paraquat	0.05	2004
206	Alpha synuclein	0.05	2004
186	Substantia nigra	0.15	2004
168	Dopaminergic neuron	0.16	2004
163	Neurodegeneration	0.04	2004
140	Environmental risk factor	0.15	2004
124	Apoptosis	0.05	2004
119	Exposure	0.11	2004
115	Dopamine	0.04	2004
115	Neurotoxicity	0.06	2004
97	Mitochondria	0.07	2004
93	Model	0.03	2004
93	Complex I	0.00	2004
89	Brain	0.01	2004
87	Rat	0.00	2004
85	Cell death	0.05	2004

Table 2 shows the top 25 keywords with strongest citation bursts. We could find out the keywords' research history and items with research potential from them. Among them, some topics burst with high strength and long duration, such as Rotenone, Pesticide, Rat Brain, and Herbicide Paraquat. The newest burst word is Nitric oxide (NO), which came out in 2006 and continued into 2013. In 1998, NO was found to be the neurotransmitter in the human body, which could keep the nervous system active and the body flexible and could relate with neurodegenerative diseases such as PD (35). NO has been studied as a signal factor in cells for 10 years, which played a vital role in cell inflammation and death of targeted neurons (36–38). However, there are no new keywords burst in recent years, suggesting that some valuable research points need to be further researched in this territory.

**Table 2:** Top 25 keywords with strongest citation bursts.

**Keyword**	**Strength**	**Begin**	**End**
Rotenone	13.3904	1986	2001
Herbicide paraquat	12.7306	2005	2012
1 methyl 4 phenyl 1	9.5699	2004	2008
Rat brain	9.2964	2005	2012
System	7.1586	2005	2012
Ethylene bis dithiocarbamate	6.5413	2004	2007
Risk factor	6.4854	2004	2009
Pesticide	6.3061	1986	2001
Lipid peroxidation	6.1932	2004	2006
Lewy body	6.0395	2004	2006
Dopamine transporter	5.7197	2004	2009
Environmental risk factor	5.4958	2004	2009
Mitochondrial complex I	5.3934	2004	2006
Striatum	5.3564	2004	2012
Tyrosine hydroxylase	5.2235	2004	2006
Parkinson's disease	4.8877	1986	2001
Substantia nigra	4.6953	2005	2006
Superoxide dismutase	4.6581	2007	2009
Combined paraquat	4.4618	2004	2006
Protein	4.0702	2005	2011
Nitric oxide	4.0247	2006	2013
Aggregation	3.9032	2004	2006
Environmental factor	3.9032	2004	2006
Mpp+	3.9032	2004	2006
Parkinson's disease phenotype	3.9032	2004	2006

### Analysis of Co-reference Articles

**Figure 3:**
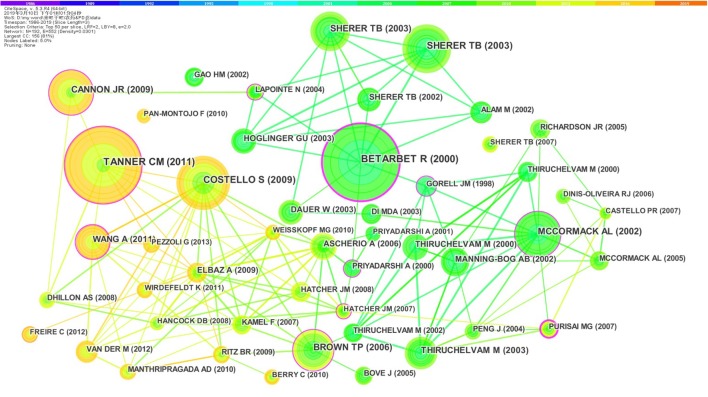
Joint mapping of co-reference references.

Many high-quality articles with innovative ideas and reliable experiment findings could be referred many times by researchers. Figure 3 shows the high co-reference articles and their collaboration. In this study, 192 references are highly cited, and the links between them reach 552 times.

In Figure 3, every circular node represents an article, and the lines that link nodes represent the co-reference. To make the picture clearer to be analyzed, this visual result only shows the references that are cited more than 25 times. Every node is composed of rings with different colors related to the cited year. The thickness of the ring depends on the frequencies of being cited, and the label of the node shows the author's name and published year. From the center of the node to the circumference, the cited years of the article are from early to late. In Figure 3, the colors of rings and lines have the same significance described above. The purple zone represents the articles cited from 1986 to 1988, the blue represents the earlier years, the green and the yellow represent the middle and later years, and the red represents 2019.

In Figure 3, the highly cited articles on the premise of node size are papers published by Tanner et al., Betarbet et al., and Costello et al. Betarbet's work was published in 2000, and had been cited as an important article many times. Although published in early years, the articles of Tanner et al., Costello et al., and Cannon et al. have been referred till now and cited frequently in recent years.

As shown in Table 3, the work of Tanner et al. has been cited the most (140 times). In a case–control study nested in the Agricultural Health Study (AHS), based on the analysis of 110 PD cases and 358 controls, the study has proved that PD was positively associated with rotenone and paraquat, which would cause mitochondrial dysfunction and oxidative stress as the pathophysiologic mechanisms of PD (39).

**Table 3:** Top 20 highly cited papers.

**Cited frequency**	**Cited reference**	**Source**	**Volume**	**Page**
140	Tanner CM, 2011	Environ Health Persp	V119	P866
136	Betarbet R, 2000	Nat Neurosci	V3	P1301-6
101	Costello S, 2009	Am J Epidemiol	V169	P919
94	Sherer TB, 2003	Exp Neurol	V179	P9
84	Mccormack AL, 2002	Neurobiol Dis	V10	P119
83	Cannon JR, 2009	Neurobiol	V34	P279
76	Brown TP, 2006	Environ Health Persp	V114	P156
73	Sherer TB, 2003	J Neurosci	V23	P10756
65	Wang A, 2011	Eur J Epidemiol	V26	P547
63	Thiruchelvam M, 2003	Eur J Neurosci	V18	P589
54	Ascherio A, 2006	Ann Neurol	V60	P197
52	Manning-Bog AB, 2002	J Biol Chem	V277	P1641
50	Thiruchelvam M, 2000	J Neurosci	V20	P9207
49	Dauer W, 2003	Neuron	V39	P889
46	Hoglinger GU, 2003	J Neurochem	V84	P491
46	Sherer TB, 2002	J Neurosci	V22	P7006
44	Elbaz A, 2009	Ann Neurol	V66	P494
42	Van der M, 2012	Environ Health Persp	V120	P340
42	Alam M, 2002	Behav Brain Res	V136	P317
40	Richardson JR, 2005	Toxicol Sci	V88	P193
40	Hatcher JM, 2008	Trends Pharmacol Sci	V29	P322

The document, made by Betarbet et al. in 2000, has been cited 136 times and ranks second. In his study, some neuropathological features of PD could be reproduced in rats exposed to rotenone through jugular vein cannulation for a long time, which induced highly selective dopaminergic degeneration in substantia nigra and the generation of fibrillar cytoplasmic inclusions that contained α-synuclein (40). According to previous studies, Sherer et al. found that chronic subcutaneous exposure to low doses of rotenone would also cause highly selective nigrostriatal dopaminergic lesions, and the aggregation of α-synuclein-positive cytoplasm in nigral neurons, which belongs to the pathological features of PD (41). His publication has been cited 94 times, ranking fourth.

The article of Costello et al. ranked third in Table 3, has been cited 101 times. The authors developed and validated an exposure assessment tool based on geographic information systems, which was used to estimate historical exposure to agricultural pesticides in the residential environment. In this case–control study, the authors investigated 368 incident PD and 341 population controls from the Central Valley of California, and found that a combination of paraquat and maneb exposure would increase PD risk, particularly in younger subjects (42). Wang et al. utilized this assessment tool as well, and he focused on pesticide use in California's agricultural central valley. This study provided evidence that exposure to ziram and paraquat would increase PD risk, and those exposed to ziram, maneb, and paraquat together experienced the greatest increase in PD risk. Wang et al.'s study was the first to implicate the role of ziram in PD etiology (43). This paper was cited 65 times, ranking ninth.

In the experiments of repeated intraperitoneal paraquat injections to mice, Mccormack et al. found that selective dopaminergic degeneration, one of the pathological hallmarks of PD, was induced, which was also a characteristic of paraquat neurotoxicity (44). His article has been referred 84 times, and ranks the fifth. The next is the work of Cannon et al., which has been cited 83 times. This study developed an improved and highly reproducible rotenone model of PD, which could replicate many aspects of the pathology of human PD and provide an insight into the pathogenesis of PD (45). The seventh is the article of Brown et al. As a review, it critically evaluated whether a relationship existed between pesticide exposure and PD. At present, there is sufficient evidence to prove an association between pesticide exposure and PD, but is not enough for concluding that this is a causal relationship (16).

It can be seen that the research on the influence of typical pesticides like rotenone and paraquat has made rapid progress in epidemiological studies, animal models, and case–control studies. However, more in-depth and innovative studies are needed to investigate the relationship between pesticide exposure and PD, especially in the further research for pathogenic mechanisms.

Based on the co-reference analysis results, this study divided the references into seven clusters through Citespace, which were described and named by log-likelihood algorithm (LLR) from the articles' indexing terms. The publications in different clusters are similar in content, extracting the labels from the articles, and the clusters' features and how they are related to each other could be clearly shown. Cluster description is shown in Table 4. The “size” is the number of publications within the cluster. The “silhouette” represents the homogeneity of a cluster, and the bigger the silhouette is, the stronger the association of articles in clusters are. The “coverage” means the consistency between the terms of article and the characteristics of cluster, so the publication with high coverage could represent its cluster accurately (26). This study selected the highest publication and summarized their research results as the description of each cluster.

**Table 4:** Clusters information.

**#**	**Size**	**Silhouette**	**Mean (Year)**	**Name (LLR)**	**Description**
0	44	0.904	2004	Pesticide-induced neurotoxicity	Review the molecular mechanism of pesticide neurotoxicity in PD model
1	42	0.618	2010	Parkinson disease	Explore the effect of typical pesticides on PD and discuss its model
2	27	0.867	2002	Rat model	Summarize the animal models of pesticide-induced PD and discuss the neurotoxic effects
3	17	0.830	2003	Pesticide exposure	Summarize the susceptibility studies of occupational exposure to pesticides
4	12	0.874	2014	Underlying pesticide exposure	Establish the mechanism of quantitative assessment of PD risk in pesticide exposure
5	7	0.966	2010	Rotenone-based modeling	Study the pathogenesis of rotenone-induced neuronal cells
6	7	0.988	2009	Intragastric administration	Study the lesions caused by pesticides in the intestinal nervous system

Cluster #0 is the largest cluster, labeled “Pesticide-induced neurotoxicity”, and contains 44 publications. The publication with the highest coverage in this cluster is by Franco R., which reviews the molecular mechanisms involved in the neurotoxicity of pesticides with an emphasis in the induction of neuronal cell death by paraquat as a model for PD (46).

In cluster #1, the article of Berry C., which analyzes the relationship between paraquat exposure and PD and discusses the limitations of chemical PD model, owns the highest coverage (47). The publication by Uversky V.N. with the highest coverage of cluster #2, focusing on the mechanisms of neurotoxicity, summarizes the toxin-induced PD models and amplify the pesticide-induced PD models (48).

The work of Paolini M. could represent cluster #3, offering a series of suggestions for susceptibility studies that focused on PD risk in population persistently exposed to typical pesticides (49). The mean published time of articles in cluster #4 is 2014. The article with the top coverage is that by Cao F.J. This review tried to establish a mechanism framework for calculating PD risk by sorting out details on transcriptome responses to PD-associated pesticide exposure (50).

The top 2 papers with the highest coverage in cluster #5 are published by Emmrich et al. and Xiong et al. Using glial cultures prepared from rat cerebella, the former investigated the effect of microglia to neuronal cell death induced by low concentrations of rotenone (51). The latter study aimed at investigating the contribution of autophagy in the pathogenesis of rotenone-induced models for PD. The results showed that autophagy attenuates the rotenone toxicity and possibly represents a new subcellular target for treating PD (52). The last cluster could be represented by Pan-Montojo et al.'s work. Researchers treated mice with low doses of chronic and intragastric rotenone in order to explore the pathogenesis of rotenone in the enteric nervous system (ENS), the dorsal motor nucleus of the vagus (DMV), the intermediolateral nucleus of the spinal cord, and the substantia nigra. The findings suggested that pesticide could reproduce the neuroanatomical and neurochemical features of PD (53).

**Figure 4:**
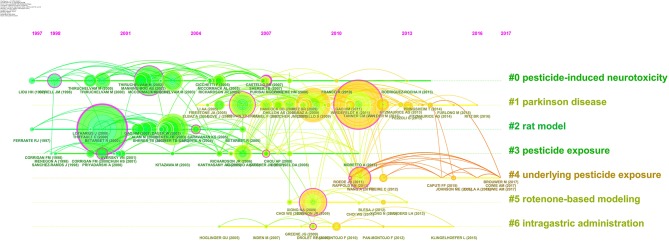
Timeline view of the clusters.

Through the analysis in clusters of co-references, we could understand the hot spots and the cutting edge of this study. The labels of cluster #0 and cluster #5, pesticide-induced neurotoxicity and rotenone-based modeling, represent the research focus in a certain extent. The kinds of highly cited references, the number of literatures, their referred situation, and the active period of each cluster are obviously different. However, Figure 4 shows the timeline visualization with Citespace that could display these differences clearly (54). Seven clusters, with citation distributions and group names, are shown in Figure 4.

Citespace only selects the top 50 most cited articles when it measures the co-references. Consequently, the statistical results are insufficient to cover all time periods. The references shown in Figure 4 are all published from 1997 to 2017, which means that the numbers of co-references, in others' times of being cited, are not enough.

In Figure 4, from 1997 to 2017, every 3 years as a slice, the timeline was divided into eight sections. The position of the node corresponds to published time, the color of the ring represents cited time, the thickness of the ring depends on cited frequencies, and the lines between nodes represent co-references. Time goes from far to near, and the color changes from green to yellow and finally turns to orange. The rank of clusters is determined by the number of references; in Figure 4, the references published in 2001 and 2010 owns the largest number, and some big clusters could cover half of the timeline, such as cluster #0, #1, and #3. The references in smaller groups are mainly published after 2007, such as cluster #4 and #5. Besides, it could be seen that most of the highly cited references in the early period would attract large attention, but their popularity usually lasts less than 6 years. For instance, the article published by Betarbet et al. in 2000 was cited frequently before 2007, and then new published papers became the hot spots, such as the article written by Tanner et al. in 2011, which has been cited to a greater extent than the former in recent years.

### Analysis of Co-author's Country

**Figure 5:**
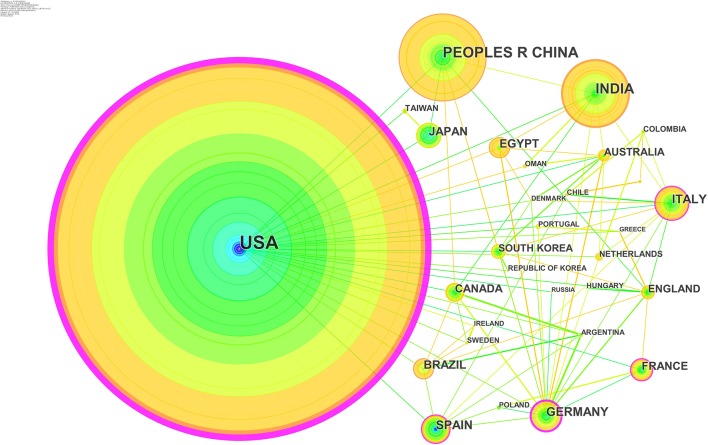
Joint mapping of productive countries.

As shown in Figure 5, nodes represent the contributing countries, lines mean the cooperation of countries, and 49 nodes and 183 links in the partner countries formed a network. The color represents different periods when the articles were published, and the color of lines follow the same rules. The size of the node composed of rings corresponds to the significance of countries toward study in pesticide-induced PD, and there is a positive correlation between the thickness of lines and the strength of cooperation. It can be seen in Figure 5 that, the United States, China, India, Germany, France, and Italy have made progress in the recent 30 years, with a steady number of publications in every period. The United States, Germany, and India cooperated with other countries frequently in this area.

The top 10 productive countries with publications are reported in Table 5. In first place, the United States accounts for 734 publications, playing an indispensable role in this field. China and India rank the second and the third, with 327 and 140 records, respectively. The number of publications in the top 4 countries is significantly different, and there is a nearly double gap in the number of publications between adjacent countries in the rank. The countries in the latter part of the list have a similar number of articles, such as Italy, Spain, and Germany.

**Table 5:** Top 10 most productive countries.

**Total publications**	**Countries**
734	USA
327	China
140	India
72	Italy
65	Spain
61	Germany
54	Japan
54	Brazil
51	Egypt
49	France

### Analysis of Co-author's Institutes

**Figure 6:**
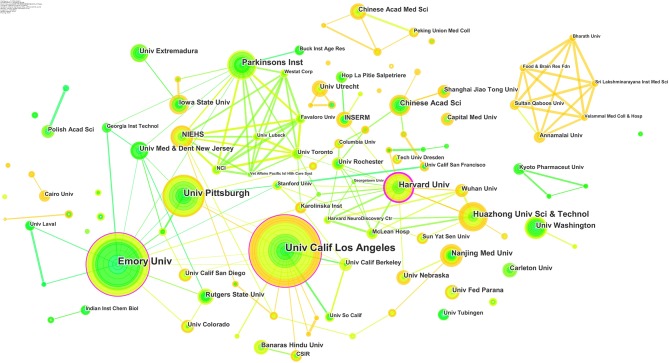
Joint mapping of productive institutes.

The analysis of all publications found 252 research institutes, and the findings and cooperation are shown in Figure 6. From the nodes and lines, it could be seen that American institutes usually cooperate with the institutes in China, Canada, and Germany from 2007 to 2016. In addition, India domestic agencies have done research together frequently since 2013.

The top 10 productive institutes are listed in Table 6. The University of California in Los Angeles ranks the first with 44 publications, followed by Emory University and Pittsburgh University. While the number of publications of other institutes is similar, the gap of the top 3 agencies increases apparently. Evidently, the major research institutes come from the United States, which have made a great influence in the research field of pesticides associated with PD. In addition, there are also some Chinese and Indian agencies making progress continuously.

**Table 6:** Top 10 most productive institutes.

**Total publication**	**Institute**
44	Univ Calif Los Angeles
38	Emory Univ
26	Univ Pittsburgh
18	Parkinson Inst
18	Huazhong Univ Sci & Technol
17	Harvard Univ
14	NIEHS
14	Nanjing Med Univ
14	Univ Washington
13	Chinese Acad Sci

### Analysis of Co-reference Authors

**Figure 7:**
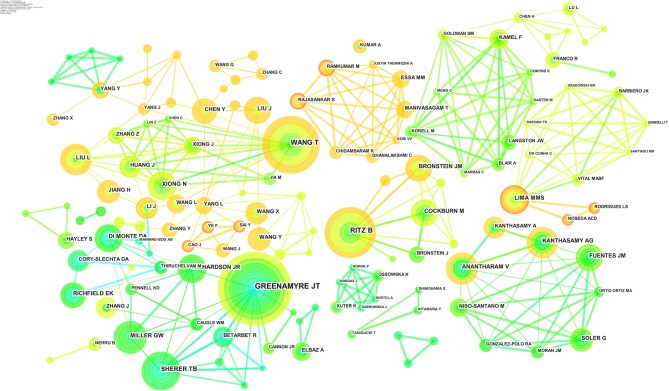
Joint mapping of productive authors.

In Figure 7, 345 authors are included in the analysis, and the lines of cooperation reach 789. The quantity of published papers is correspondingly proportional to the size of nodes, and the color of ring is dependent on the time of publication. The lines between nodes represent the cooperation, whose thickness is related to the extent. It can be seen that, since 2001, Wang et al., Ritz et al., and Liu et al. have been creating articles in almost every period. As for some authors with high yield like Greenamyre et al. and Sherer et al., their works were mainly published from 1998 to 2007. Moreover, as a result of the shades of orange and red node nets, many Chinese researchers such as Wang et al. and Yang et al. have been active since 2007, and they usually cooperate with domestic scholars. Indian researchers like Manivasagam T. and Rajasankar S. cooperated with each other frequently after 2016.

Table 7 lists the top 10 productive researchers. Apparently, the top 3 researchers have a clear lead in publication, but the number of other authors is similar. The most productive researchers come from America. Greenamyre et al. began his work in the field in 1998 and has been productive until now. In the early period, he mainly studied the pathogenic mechanism of rotenone-induced PD animal models. After that, he continued to explore the relationship between environmental toxic and neurodegenerative disorder and has created many valuable overviews with some researchers and began to focus on the combined effect of pesticides and genes in PD pathogenic mechanism. Mainly published after 2011, the papers of Wang et al. turned up in 2009. For Ritz et al. beginning in 2000, many of his findings originated from the epidemiology of pesticide exposure, including studies on paraquat, maneb, and organophosphates. In recent years, he began to pay attention to the combined influence of pesticides and genes toward PD risk as well.

**Table 7:** Top 10 most productive authors.

**Total publication**	**Author**
42	Greenamyre JT, 1998
32	Wang T, 2009
29	Ritz B, 1998
22	Sherer TB, 1998
18	Liu L, 2013
18	Miller GW, 2004
18	Kanthasamy AG, 2001
17	Xiong N, 2009
17	Lima MMS, 2010
16	Di Monte DA, 2001

### Analysis of Co-reference Journals

The co-citation of journals analysis can show their contribution to this field, and the highly cited journals are usually suitable for submitting articles. Table 8 lists the top 10 journals, all of whose cited levels are high, and the highest one is *Journal of Neurochemistry*, which has been referred 646 times. *Neurotoxicology* has been cited 463 times, ranking 10th. Among them, the journals in research field of neurology, biology, and medicine occupied the main status, which is consistent with PD and its pathogenic mechanism.

**Table 8:** Top 10 highly cited journals.

**Cited frequency**	**Journal**
646	J Neurochem
609	J Neurosci
581	J Biol Chem
574	Brain RES
529	Neurology
489	ANN Neurol
488	P Natl Acad SCI USA
472	EXP Neurol
469	Neurobiol DIS
463	Neurotoxicology

## Discussion

In this study, based on the research topic of pesticide exposure and PD, we analyzed 1767 articles in WOS core collection and PubMed, through various statistical items such as references and keywords in Citespace, and gained solid conclusion from the analysis of visualization.

The number of publications about pesticide-induced PD has increased continuously since 2000, and its speed is slow without burst year, which shows the steady development of this field. Analyzing the literature sources, the United States, situated in a leading position in this research, has published more than 700 literatures in the past. Lots of indispensable agencies and authoritative researchers come from America. Many innovative experiment models, research methods, and advanced findings originate from their works. Moreover, China also plays a crucial role in the research field with 327 articles published in total, and Chinese researchers have been active in the recent 10 years. The supplement to the traditional literature reviews based on personal judgment and interpretation (32), typically, could help researchers understand the characteristics of research collaboration in this research field. Readers could identify the major scholarly communities and the most cited scholars and journals in the field, which may help them seek for suitable research collaborators (55).

On the account of the analysis of the keywords, which can be seen from the highly cited references, the verification of correlation between pesticide exposure and PD has become mature, with the appearance of pesticide-induced PD model and the development of the relevant epidemiologic study, especially for rotenone and paraquat, which have been studied as the typical pesticides frequently. Specifically, researchers could learn about the possible types of pesticides inducing PD from the analysis of co-occurrence keywords, and they could also find the deep reasons and molecular mechanisms of various pesticides inducing PD through the high co-reference articles (30). Identifying the clusters of co-reference, “Pesticide-induced neurotoxicity” is the largest group, and other items such as “Rat model,” “Rotenone-based modeling,” and “Intragastric administration” also take up the main parts. It shows that the pathogenic mechanism in molecular aspect is the hot spot.

According to the analysis above, we make a prediction on the future research direction as follows. Firstly, as shown in Tables 1, 2, the research on rotenone and paraquat inducing PD has been hot and mature in the past (56–58), despite being deficient for other pesticides, such as pyrethroids, organochlorine, and organophosphate pesticides (59–62). Based on the findings and advanced methods, the study on the association between other kinds of pesticides and PD would increase in the future. Secondly, it is widely accepted that PD is induced by the combination of various factors, and the projects on a single sort of factor have reached a certain amount (14, 63–65). In the future, it is believed that the combined effect such as pesticides and genes, different kinds of pesticides, and pesticides and lifestyle will attract increasing attention. Till now, the combination of paraquat and maneb has been put forward and put into use many times (24, 38, 42, 43, 66, 67). Thirdly, the pesticide-induced PD models are less well established, and almost all of them are rotenone-induced PD models and PD models induced by paraquat combined with maneb (45). As the development of technology, there is an increasing number of pesticide-induced PD models that might turn up. Besides, the assessment method of pesticide exposure and the verification of PD will step forward as well (68–72). For example, Yoon I.S.' team has developed and validated a sensitive and reliable assay with neuroblastoma SK-N-SH cells in order to screen for small molecules to reverse rotenone-induced neurotoxicity (68).

The results of this study depend on literature screened from WOS core collection and PubMed, and the effort is to refine scientific and effective analysis results from the proprietary document library. However, the source of literatures and the subjective factors will give rise to the difference in the quality and quantity of articles. Moreover, different parameter settings in Citespace will also change the analysis result of visualization. Therefore, there are some limitations in this study, as it can only analyze the current situation and the trends of research in pesticide exposure inducing PD from limited view. Hence, we will keep on conquering and improving these shortages, making the trend prediction more precise in future research. Moreover, although having been screened, these articles searched might be more associated with medical or pharmaceutical studies, and the relevance to environmental issues or pesticides may not be enough. In the future, we need to be more specific in the aspect of environment.

## Conclusion

Based on the research topic of pesticide exposure and PD, this study carried on the scientometric analysis from the keywords, cited references, countries, authors, and some other aspects by using Citespace. In this research field, most productive institutes and authors come from America and China, and the high frequency of “Rotenone” and “Paraquat” shows that they are hot spots. Furthermore, according to the visual analysis results and critical reading of highly cited references, we make a prediction on the future research direction. The study on the association between new kinds of pesticides and PD will increase in the future, and the research trend of pathogenesis will shift from single factor to multiple factors. In addition, more pesticide-induced PD models will turn up, and new assessment methods of pesticide exposure and the verification of PD will step forward as well.

## Data Availability Statement

The raw data supporting the conclusions of this article will be made available by the authors, without undue reservation, to any qualified researcher.

## Author Contributions

CL contributed to organize the study, prepared datasets, performed the statistical analysis, and drafted the manuscript. ZL conducted study design, performed the statistical analysis, and drafted the manuscript. ZZ contributed to organizing the study, prepared datasets, and revised the manuscript. YL contributed to study design and interpretation of analysis. RF contributed to study design and dataset preparation. FL and JZ contributed to dataset preparation and revision of the manuscript. All authors read and approved the final manuscript.

### Conflict of Interest

The authors declare that the research was conducted in the absence of any commercial or financial relationships that could be construed as a potential conflict of interest.
